# Supplementary material for: Are carbon emissions trading and green financial instruments synergistic? -Comprehensive quantitative research based on content analysis
Source: PLoS One. 2024 Mar 7;19(3):e0298601. doi: 10.1371/journal.pone.0298601 (PMC10919601; doi:10.1371/journal.pone.0298601)
Supplement: S1 Data — (ZIP) [file pone.0298601.s001.zip › Data/地级市Z_F工作报告.zip!/╜¡╦╒/╬▐╬2╩╨/└┤╫╘╣1⁄2╓┌║┼í╛┬φ┐╦╩2╛▌═°í┐_2012.docx]

各位代表：

　　现在，我代表无锡市人民政府，向大会报告工作，请予审议，并请市政协委员和其他列席人员提出意见。

　　2011年的工作回顾

　　2011年是宏观经济环境发生较大变化的一年。面对严峻复杂的经济形势，在中共无锡市委的领导下，全市上下以科学发展观为指导，认真贯彻中央和省的各项决策部署，团结一致，攻坚克难，经济社会发展取得新的成绩，全面完成了市十四届人大四次会议确定的各项任务。预计全市地区生产总值增长11.5%；财政总收入同口径增长12%，其中一般预算收入增长20.1%；城镇居民人均可支配收入、农民人均纯收入分别增长13%和16%；城镇登记失业率2.6%；居民消费价格指数105.1，“十二五”的发展实现了良好开局。

　　(一)全力以赴推动发展，经济保持平稳运行

　　工业保持平稳增长。积极应对宏观环境变化，突出重点狠抓薄弱环节，及时采取加强运行调控、加大市场开拓、扶持企业发展、扩大有效投入等一系列措施，促进工业经济的稳定增长。预计全市完成规模工业增加值3350亿元，增长11.2%。农业现代化水平提高。新增高效农业面积5.6万亩，高效农业占耕地面积比重达54.6%。新增设施农业面积2.9万亩，农业适度规模经营面积比重达78.8%。太湖外向型农业示范区、阳山水蜜桃科技园被认定为省现代农业产业园区。预计全市现代农业发展水平达74.8%。村集体资产总额410亿元，预计村均可支配收入540万元，增幅超过10%。投资结构逐步优化。鼓励和引导民间投资，扩大新兴产业和优势产业投资比重。预计全市实现固定资产投资3169亿元，增长18%；其中，民间投资增长31.8%，服务业投入增长19.1%。投资5亿元以上的在建产业项目达160个左右。IBM　技术中心、清华应用技术研究院等落户我市，海力士四期技术升级、威孚工业园、上汽商务车等重大项目加快推进，联通物联网、航天天基网、中电科IC中心等项目加快启动。消费市场基本稳定。改善居民消费环境，完善城乡商贸流通体系，预计完成社会消费品零售总额2117亿元，增长17%。粮食科技物流中心竣工投用。全力保障市场供应，强化价格监管，建成市属4万亩蔬菜基地和109家平价商店(蔬菜类)，及时实施肉菜供应应急方案并落实采购储备，物价涨幅低于全国全省平均水平。开放型经济逆势求进。预计实现到位注册外资35亿美元，引进重大项目46个，服务业利用外资占比达47.4%，新兴产业占制造业利用外资比重达40.6%。加快调整外贸产品和市场结构，强化出口品牌建设，预计完成进出口总额700亿美元，增长15%以上；其中出口420亿美元，增长15%以上。新批境外投资项目75个，境外投资额翻一番。柬埔寨西港特区建设列全国6个境外合作区前茅。国际友好城市增加到40个，荣获全国“友城战略发展奖”。江阴高新区升级为国家级高新区，锡山经济开发区升格为国家经济技术开发区，锡通科技产业园建设全面启动。对台合作不断拓展。金融支撑作用加强。深入开展银企对接，加大对经济发展的支持，预计全市银行本外币存贷款余额分别新增912亿元、805亿元，实体经济新增贷款占比达86%。创新金融产品与服务，银行表外、发债、上市等社会融资总量达1655亿元。科技金融建设深入推进，新设创投机构39家，新增基金规模122亿元，农银150亿元无锡股权投资基金正式注册成立。科技支行、中小企业专营机构、科技小贷等作用逐步显现，新增高新技术企业贷款53亿元。新增上市公司9家，融资78亿元。花旗银行、恒丰银行、浙商银行等一批境内外银行机构落户无锡，中外合资华英证券正式营业。要素保障力度加大。建立市本级土地调控机制，完成大规模土地整理8.2万亩，国家资源的争取实现重大突破，一批重点工程和重要项目用地得到保障。实施有序电力供应计划，有效保障了生产和城市生活需求。

　　(二)加大结构调整力度，产业竞争力不断增强

　　创新资源集聚效应放大。以完善技术创新体系为核心，推进创新体系建设，实施科技成果转化工程，预计高新技术产业实现产值5350亿元，同比增长20.8%，占规模以上工业总产值比重达36%。区域创新能力不断提高，预计全社会研发投入占地区生产总值比重达2.6%，获省重大成果转化项目立项累计124个，建立省级以上企业工程技术研究中心252家，均列全省第一。加快创新国际化步伐，与美国麻省理工学院成功签订合作协议，中以(无锡)国际技术转移中心和国际科技投资转移公司顺利落户。“人才特区”建设加快，新落户“530”项目480多个，年销售超千万元的“530”企业超过60家。实施产业升级行动计划。进一步聚焦新兴产业和重点产业领域，着眼做强微电子、光伏、环保、重大装备制造、旅游等五大优势产业，着力培育物联网与云计算、工业设计、文化创意、生物医药、软件与服务外包等五大新兴产业，分别进行专项部署，建立专门工作队伍，使抓手明确、措施到位。强化产业升级导向，健全重大项目联席会议制度，赴美、日、香港及京沪深等地成功开展招商，项目建设在全市进一步形成氛围。把物联网发展作为引领全市转型升级的突破口，推动由资源集聚加快向应用推广和产业化转变，物联网博览园改造工程启动，中国传感网国际创新园二期正式开园，感知交通、环保、卫生、家居、农业等国家级应用示范项目全面展开。先进制造业层级提升。实施制造业竞争能力提升工程，加快构建以技术支撑、高端引领、特色发展、品牌竞争为特征的发展新格局。新增国家驰名商标11件，省著名商标55件，省名牌产品24个，预计营业收入超百亿企业增加到25家，分别有15家和23家企业入围中国企业500强和中国制造业500强，分别占江苏省入围企业的30.6%、45.1%。远东电缆荣获全国质量奖，红豆集团、长电科技被授予第四届市长质量奖，法尔胜集团成功接管钢丝绳国际标准化组织，无锡跻身中国十大品牌城市行列。加快布局结构调整，光电新材料科技园等一批改造提升项目加速推进。服务业发展势头强劲。改善服务业结构层次，实施服务业发展超越工程，预计服务业增加值占地区生产总值比重达44%。广播影视、创意设计、动漫游戏等文化产业快速发展，无锡动漫公共技术服务平台被列入全国十大公共技术平台，无锡(国家)数字电影产业园启动建设，华映文化产业基金正式成立。新增全球服务外包100强、国内50强投资企业4家，“123计划”企业17家，服务外包离岸执行金额23.8亿美元，增长53%，列全省第一。加强旅游资源开发，预计实现旅游总收入890亿元，增长18.9%，全年接待入境旅游者突破百万人次。

　　(三)加快生态宜居城建设，城乡现代化建设扎实推进

　　城市功能有效提升。高标准建设太湖新城，无锡大剧院、金融商务第一街区、太湖国际博览中心二期等项目有序推进。锡东新城核心区路网体系和交通枢纽功能基本形成。综合交通体系加快构建，无锡中央车站、京沪高铁无锡东站正式启用，宁杭城际铁路无锡段完成主体工程。地铁1号线建设加快推进，2号线全面开工。太湖大道、高浪路、金城东路快速化改造等工程竣工投用，市区建成城市道路106.5公里，建成农村公路157公里、桥梁185座。机场新辟无锡至台北等客货运航线13条，客货运吞吐量分别增长16%。苏南运河无锡段三级航道整治工程全线建成通航。港口货物吞吐量完成2亿吨。实施京沪高铁沿线整治，加强城市管理和环境综合整治，市容市貌得到改善。节能减排完成良好。推进合同能源管理和感知能效服务平台建设，实施一批重点节能减排和循环经济项目，加快淘汰落后产能，深入开展“三高两低”企业整治，节能减排完成省下达的目标任务。加快推进中瑞低碳生态城建设，无锡被评为2011中国自主创新十大低碳城市。太湖治理扎实推进。加强太湖调水引流和监测预警，及时开展蓝藻打捞、生态清淤，实现了太湖安全度夏和水利汛期的安全度汛。开展集中式饮用水源地规范化整治，饮用水源地水质达标率100%。完成893块排水达标区创建，达标区域基本覆盖主要建成区。加快推动“清水流域”示范工程建设，完善区域环境监测体系。推进农村地区“三集中”，高标准完成宜兴、锡山农村环境连片整治，抓好农业面源污染治理。生态工程建设成效明显。金城湾公园建成开放，蠡湖地区环境综合整治工程全面竣工。积极推进“绿色无锡”建设，完成造林绿化面积5.9万亩，新增游园绿地38个，预计林木覆盖率25%。大力开展湿地保护与恢复，长广溪湿地公园一期、九里河湿地公园等相继竣工。成功举办第二届中国湿地文化节暨亚洲湿地论坛，梁鸿湿地被授予国家湿地公园称号。

(四)切实办好民生实事，群众生活水平持续提高

　　居民收入稳定增长。实施居民收入倍增计划，城镇居民人均可支配收入、农民人均纯收入分别为31350元、16250元。市区企业职工退休金人均增加171元，居民养老保险基础养老金标准提高到每月230元，市区低保标准由420元调为460元。扩大失业保险基金支出范围，市区财政支出社保补贴和岗位补贴2.8亿元，惠及下岗失业人员21.9万人次。社会就业稳步扩大。健全城乡统一的人力资源服务体系，扎实做好农村转移劳动力、就业困难人员、退役军人等重点群体就业工作。预计本地农村劳动力充分就业率提高到94.2%。大力开展各类就业创业培训，预计全市新增城镇就业12.6万人，城镇失业人员再就业9.7万人，就业困难人员实现就业3.4万人。社会保障不断完善。在稳定扩大社会保障覆盖面基础上，着力提高社会保障标准。全市企业职工养老、医疗、失业、工伤和生育保险分别扩面12.8万人、22.4万人、13.3万人、20.7万人、12.6万人，城乡基本社会保险覆盖率达98.8%以上。加快推进南山慈善家园、滨湖区社会福利院等项目建设，投资2.5亿元兴建市失能老人和重度残疾人托养中心。年内新增机构养老床位3204张，每百名老人拥有机构养老床位数达2.5张。尊老金发放范围下限延伸至80周岁，实现高龄老人全覆盖。创新居家养老服务模式，新增居家养老服务点337家。我市位列全国慈善城市综合指数第四名。居民住房保障有力。瑞星家园二期、惠景家园等共60万平方米保障性住房竣工交付使用。全市新增公共租赁住房17109套，拆迁安置房竣工670万平方米，新开工经济适用房7004套。全市享受廉租房政策居民5857户。市区旧住宅区整治改造完成97万平方米。完成背街小巷整治120条。积极贯彻国家房地产调控政策，房地产市场运行总体平稳。为民办实事成效显著。11件为民办实事项目全面完成。市区更新老旧自来水管网110公里，完成自来水水表出户6663户。市区新增天然气用户9.4万户，新建汽车天然气加气站4座。市区人防工程竣工32万平方米，人防工程社会利用率大幅度提高。市区新增公交营运车辆85辆，新辟公交线路10条，建成乘客公共出行信息导乘查询系统。扩大免费乘坐市内公交残疾人范围，预计受惠2.5万人。实现全市域镇村公交全覆盖。办理法律援助案件4389件，“12348”受理群众法律咨询2.1万余人次。我市荣获2011中国最具幸福感城市。

　　(五)推动各项事业全面进步，社会保持和谐稳定

　　教育事业优先发展。完成44所幼儿园的新建、改扩建，60%以上的村幼儿园建成市优质园。完成中小学校舍安全工程加固66.5万平方米，重建23.7万平方米。改造提升63所义务教育相对薄弱学校，新增11所市义务教育现代化学校。江南大学太湖学院转设为独立建制的无锡太湖学院。江南大学无锡医学院开始筹建。医疗卫生稳步提升。顺利通过国家卫生城市复查，继续加强卫生村镇创建工作，11个镇通过国家卫生镇全国复审。完成医药卫生体制改革三年阶段性目标，87家社区卫生服务中心、718家社区卫生服务站全面实施国家基本药物制度并实行零差价销售，实现市域范围全覆盖。公立医院改革迈出新的步伐。文化事业扎实推进。大力推进公共文化设施“全年无休”免费开放服务。滨湖区、崇安区、北塘区文化馆、图书馆和新区图书馆等一批公共文化设施建成开放。农家书屋增至776个并实现行政村全覆盖。全市国家级非物质文化遗产名录项目增至9家。宜兴市被评为国家历史文化名城。中国泥人博物馆建成开放，惠山老街获“中国十大历史文化名街”称号，清名桥历史文化街核心区全面建成。成功举办第27届亚洲邮展，城市知名度进一步提高。体育事业协调发展。开展“幸福进万家，体育健万民”行动，无锡被列为全省唯一的“全国全民健身示范城市”试点城市。体育场馆的管理运营水平不断提高。新增公共体育设施面积52.2万平方米，人均2.36平方米。经常参加体育锻炼人数比例超53%，名列全国前茅。社会管理进一步加强。强化社区工作站的基础管理作用，全市城乡社区扁平化管理基本实现全覆盖，50%的社区达到人口综合服务示范社区要求，流动人口服务管理得到新的加强。及时部署实施安全生产隐患排查和专项行动。强化食品安全监管，进一步加强餐厨废弃物管理。争创双拥模范城六连冠工作进展顺利。深入开展矛盾排查化解、信访疏导化解、突发事件应急处置等工作。切实加强社会治安防控体系建设，大力开展治安问题专项整治，社会总体保持稳定。

　　(六)科学统筹政府工作，服务型政府建设得到加强

　　依法行政水平进一步提高。加快构建权力运行监控机制，市级部门基本建成内控制度体系，三级行政权力网上公开透明运行实现全覆盖，无锡网上(感知)监察系统建成并投入运行。实施新一轮公务员能力培训，强化公务员依法行政能力。深入推进“法治无锡”、“平安无锡”建设，我市被评为全国法制宣传教育先进城市。作风与效能建设进一步加强。积极开展创先争优活动，政府服务发展、干部服务群众、机关服务基层的意识更加自觉，攀高比强、创新实干的氛围逐渐浓厚。严格落实廉政建设责任制，加大廉政准则执行力度。“三公”等重点领域突出问题专项治理取得积极进展。继续推进民主评议，民主评议基层站所和行政执法部门活动共为群众解决问题2964个，群众对评议部门的平均满意率达95.4%。公共服务能力进一步增强。取消行政事业性收费31项。推行经营性服务收费项目目录管理，全面清理中介服务收费和经营性服务收费，制定出台工商咨询服务收费项目及标准，收费标准平均下降30%。圆满完成第六次人口普查。加强预算资金管理，完成专项资金预算和支出评估论证，开展公共资金绩效审计。市公共资源交易服务中心建成投运。开通“12345”公共服务热线，共受理群众诉求17.8万个，办结率94.4%。市政府门户网站列中国政府网站评估第一。坚决贯彻市人大及其常委会的决定决议，认真办理人大代表的建议、意见和政协委员提案。史志、档案、侨务、人武、民族宗教、气象、无线电管理、防震减灾等工作取得新成绩，妇女、儿童、老龄、残疾人等各项事业有了新的发展。

　　各位代表，刚刚过去的2011年，是无锡发展不平凡的一年。我们在应对困难和挑战中取得了超出预期的成果，同时也探索了在复杂环境中推动科学发展的有益实践。过去一年，我们坚持把握大局，努力将贯彻上级精神与无锡实际相结合、将谋长远与抓当前相衔接，紧紧围绕落实“六个注重”、实施“八项工程”及率先基本实现现代化的战略部署，有力组织实施我市各项行动，大力推进无锡“两个率先”的历史进程，同时一着不让地对当年工作作出计划安排，奋力完成年初确定的目标任务，在多重困难条件下实现了经济社会的较好发展，为全年发展奠定了坚实基础；我们坚持科学统筹，妥善处理好经济与社会发展的各项关系，把稳增长作为无锡发展的首要环节和当务之急，把促转型作为应对市场变化的关键举措和突破口，把惠民生作为政府工作的根本追求，把保稳定作为经济社会发展的重要保障，加大解决突出问题的工作力度，不仅有效应对了即期遇到的困难与挑战，而且还积极化解了过去遗留的一些矛盾问题，为以后发展赢得了主动；我们坚持群策群力，鼓励和支持基层创新，广泛调动各方面的积极性和创造性，依靠群众共谋发展，各地、各部门单位及社会各界，自觉服从大局、服务发展，作出了积极贡献，全市上下形成迎难而上、团结奋进的强大合力。过去一年取得的成绩来之不易，凝聚着全市广大干部群众的智慧和力量，是全市人民共同奋斗的结果。在此，我代表无锡市人民政府，向全市人民，向人大代表、政协委员，向离退休老同志，各民主党派、工商联和无党派人士，各人民团体，驻锡解放军、武警官兵和公安干警，国家和省驻锡单位，以及关心和支持无锡建设发展的海内外朋友，表示衷心的感谢和崇高敬意！

　　在肯定成绩的同时，我们也清醒地看到，我市发展还存在许多突出的矛盾，经济运行面临着不少新情况和新问题。经济增长的后劲不足，提速增效压力增大，部分企业生产经营困难，发展不可持续的矛盾还比较突出；产业层次和整体水平不高，自主创新能力不强，重大产业化项目缺乏，经济增长与产业转型呈现双重压力；中心城市辐射带动力不足，各板块功能集聚、错位发展水平还有待提高；资金、土地资源等对发展的制约日益突出，环境承载能力还比较脆弱；一些多年积累性的矛盾逐步显现，维护社会稳定的压力加大。同时，政府工作也面临着许多新的课题，作风建设有待进一步转变，反腐倡廉建设还需要进一步加强。对此，我们必须高度重视，采取积极有效的措施切实加以解决。

　　2012年工作的总体要求

　　2012年是实施“十二五”规划、推进率先基本实现现代化建设的关键之年，做好今年工作至关重要。当前，世界经济增长放缓，国际贸易回落，金融市场动荡，世界经济严峻复杂，各类风险和不确定因素增多。国内要素瓶颈制约加重，市场需求萎缩，区域间的竞争加剧，无锡发展正面临着前所未有的挑战与考验。我们必须保持清醒头脑，做好充分应对各种困难挑战的思想和工作准备，既要深刻认识宏观环境的复杂性和发展的艰巨性，又要准确把握无锡发展所具有的优势条件，勇于难中求进，善于危中寻机。经过多年建设，无锡已形成了良好的发展基础，拥有应对市场变化的竞争实力和实践经验。只要我们保持和弘扬“四千四万”的优良传统，审时度势，乘势而上，就一定能够战胜困难，开启率先基本实现现代化的新的局面。

　　今年政府工作的总体要求是：坚持以邓小平理论和“三个代表”重要思想为指导，全面落实科学发展观，以率先基本实现现代化为目标，坚持稳中求进、好中求快、又好又快，着力加快经济转型升级，着力加强科技创新，着力推进城乡建设，着力深化改革开放，着力推动民生改善，保持经济平稳较快发展，保持社会和谐稳定，以经济社会发展新的优异成绩迎接党的十八大胜利召开。

　　综合考虑各方面因素，今年经济社会发展主要预期目标是：地区生产总值增长11%左右；地方财政一般预算收入同口径增长12%以上；固定资产投资增长16%以上；社会消费品零售总额增长17%以上；节能减排完成省下达指标；城镇居民人均可支配收入增长12%以上，农民人均纯收入增长13%以上；城镇登记失业率控制在3.5%以内；居民消费价格指数控制在省定范围内。

各位代表：无锡已开启“第二个率先”的伟大征程。跨入新的一年，市委十二届二次全会提出了“当好率先基本现代化先行军和科学发展排头兵”的任务要求。我们要进一步发挥优势，抢抓机遇，以饱满的精神状态、明晰的工作思路和更加务实的发展举措，更大力度推进经济社会又好又快发展。一是立足率先发展，在各项工作上迈出新步伐。切实把握今年工作的总基调，坚持以率先争先确立发展目标，着眼于能好则好、能快则快，全力推动经济的平稳较快增长，实现经济综合实力的进一步提高。同时，全面推进各项事业，努力使无锡的发展保持领先，在全省走在前列。二是着眼创新发展，在体制机制建设上增创新优势。坚持创新驱动、人才引领，大力提高科技创新的产业化程度，通过创新增强核心竞争力。把技术创新与制度创新结合起来，通过改革推进体制机制创新，激发经济增长的新的动力和全社会创新创造活力。三是加快转型发展，在优化升级上实现新突破。大力支持实体经济发展，聚焦产业转型，坚持新兴产业扩张与传统产业提升“双向并举”、先进制造业和现代服务业“两轮驱动”，实现实体经济的水平提升。注重城乡统筹，推动一体融合发展，提升城市品质，改善农村面貌，推进城乡在转型中加快发展，在发展中实现转型。四是促进和谐发展，在服务民生上取得新成效。坚持以人为本的政府工作导向，建立健全民生诉求表达、民生问题解决和民生投入保障机制，提高民生保障水平和“幸福无锡”建设的群众满意度。加强和创新社会管理科学化水平，夯实基层基础工作，积极预防和化解社会矛盾，不断营造和谐稳定的社会环境。

　　2012年的工作任务

　　实现今年发展目标，要突出重点，切实抓好以下主要工作：

　　(一)推动经济稳定增长，确保经济持续健康运行

　　抓住发展不动摇，积极破解难题，确保主要经济指标平稳较快增长。强化经济运行调节。加强对宏观经济走势的跟踪分析和预测预警，及时解决经济运行中的苗头性、倾向性问题。加强生产要素供给，积极组织煤电油气调配，着力保障重大产业项目和重点民生项目资源需求。广泛拓宽融资渠道，鼓励和支持在锡金融机构更多争取国家信贷资源投放，确保新增贷款增速不低于全省和苏南平均水平，各类融资总额比上年有所增长。支持实体经济发展。落实推动先进制造业、现代服务业和中小企业发展的一系列政策措施，为实体经济成长壮大创造良好环境，鼓励和引导人才、资金等要素更多投向实体经济，积极组织工业企业大力开拓目标市场，促进产销对接，努力增加无锡产品市场份额。加大对企业扶持力度。扎实开展各级政府领导与企业挂钩联系服务活动，主动为企业排忧解难。全面排查和清理企业收费项目，切实减轻企业负担。鼓励企业“稳心留根”，大力支持新办企业，促进符合产业发展方向的企业加快发展。抓住市场变化机遇，鼓励企业加快兼并重组。优化中小企业金融服务，积极发展科技金融服务载体。加快企业上市培育步伐。扩大市场消费需求。实施商贸流通提速计划，支持商贸服务业龙头企业做大做强。吸引国内外知名商业巨头在无锡投资布点，提升三阳广场、胜利门、河埒口、太湖广场等商圈规划建设水平。扩大网购、信用消费和循环消费，培育更多新的消费热点。鼓励开发使用本地新产品，加大产品推广应用。实施“光伏屋顶”计划，促进太阳能光伏产品的市场应用和节能减排。支持房地产投资开发，进一步完善鼓励消费的政策措施，促进房地产市场平稳健康发展。

　　(二)大力实施创新驱动，加快产业转型升级

　　抓住市场变化的倒逼机遇，不失时机地加快结构调整，依靠科技创新推动产业转型升级，提高经济增长质量和效益。强化科技人才支撑。深入部署国家创新型城市试点工作，围绕建设“东方硅谷”目标，更大力度引进和培育高层次人才，加快推动“530”项目产业化进程。启动实施大学生引育创业五年行动计划，建立较为完整并具有吸引力的人才政策体系，促进形成各类人才集聚无锡的良好氛围。强化企业创新主体。积极完善区域创新体系，推动开放创新，着力提升企业创新能力，鼓励企业参与国际、国家标准的制定。重点建设物联网研究院、国家光伏产品检测中心、集成电路设计中心等科技公共服务平台，实现大中型工业企业研发中心全覆盖。市级财政每年投入20亿元资金，以引导和推动科技创新及产业转型。全社会研发投入占地区生产总值比重达2.7%以上，高新产业产值占工业总产值比重达38%。壮大新兴产业规模。按照高技术含量、高附加值和高成长性要求，在新兴产业急需的关键领域取得一批重大技术突破，形成一批具有自主知识产权和品牌的核心新产品，培育一批具有竞争力和较大影响力的骨干企业，扩大新兴产业产出规模。支持科技型中小企业发展，鼓励中小企业与大企业开展协作配套和联合创新，打造掌握核心技术、具有较强竞争力、形成上下游配套的高端产业链条。深入推进国家传感网创新示范区建设，努力抢占制高点，加快产业化步伐。启动实施“智慧城市”计划，推进感知环保、感知交通、感知电力等一批应用示范工程，使之尽快形成产业规模。推动制造业高端发展。加快传统制造业和优势产业技术升级，提高产品档次和附加值，增强产业竞争力。全力支持企业技术改造，技改投入占工业投入比重达75%。实施100项“两化融合”重点项目，新增省级“两化融合”试点(示范)企业20家以上。推广企业卓越绩效管理模式，推进企业品牌产品规模化工程，新增省级以上品牌90个，国际注册商标40件。加快现代服务业发展。深入实施服务业发展超越工程，服务业增加值占地区生产总值比重达45%。重点培育一批软件服务外包龙头企业。开发和整合旅游资源，优化体制机制，加强营销策划和重点市场开拓，加快发展旅游综合体，重点建设旅游目的地，争取旅游服务业发展较大突破。积极引进国内外知名会展企业，打造具有较高水准的会展品牌。整合与建设石塘湾、新安等物流园区，积极发展口岸保税物流、空港物流、城市配送等新型业态。大力发展银行、保险、债券业务与产权市场，加快推进太湖新城金融商务街区和一批重大城市综合体建设。实现文化产业振兴。科学规划文化产业布局，加强文化人才队伍建设，重点支持影视传媒、创意设计、数字出版、动漫网游、演艺娱乐等产业发展。精心打造无锡(国家)数字电影产业园、新区创意产业园、无锡国家动画产业基地、崇安家居设计中心等集聚区，加快建设国家级文化产业示范基地。加强宜兴紫砂、惠山泥人、竹刻、锡绣等非物质文化遗产的传承与保护，加快市场价值开发和产业化。鼓励文化企业在与科技、金融、旅游等产业融合中创新业态，延伸文化产业链。推进农业现代化建设。争创国家现代农业示范区，加快发展高效设施农业，提升农田装备水平，高效农业、设施农业、高标准农田面积比重分别达56%、14%和60%。建成千亩以上市级现代农业园区10个，农业园区化比重达20%。大力发展生物农业、感知农业、生态农业，不断提升农业科技自主创新能力。加快发展农民专业合作社和为农服务示范社，提升农村合作经济水平。

　　(三)狠抓重大项目建设，加快培育新的增长点

　　把项目建设作为保增长促转型的重要抓手，开展“项目建设年”活动，加大有效投入，切实增强发展后劲。保持投资较快增长。发挥政府投资引导作用，调动民间投资积极性，安排市级重点项目140项，实施项目总投资2839亿元，年内计划投资663亿元。确保固定资产投资较快增长，其中工业投入增长12.5%，民间投资增长20%以上。突出重大项目建设。积极推进深南电路半导体封装基板研发制造基地、国电光伏太阳能电池及组件等100项重大产业项目建设，发挥重大项目带动示范作用。集中资源、聚焦重点，加快培育引进一批投资规模大、技术水平高、市场前景好、产业关联度和带动性强的重大新兴产业项目。提升招商引资水平。努力巩固已形成的良好氛围，完善三级招商网络，强化和优化招商队伍，建立健全招商激励机制，进一步加大招商引资力度。按照产业需求，重点加强终端产品和产业链招商，强化战略性新兴产业、先进装备制造业、生产性服务业等领域的项目引资。设立专项引导资金，鼓励世界500强、中国100强在无锡设立研发、销售、结算中心与地区总部。优化项目服务机制。强化项目评估，完善《重大产业项目评估实施办法》，引导提高项目投入强度和亩均产出效益。建立项目招商协调机制，加强市级统筹，发挥板块作用，实现全市联动招商。在对项目实行“三级”评估基础上，对重特大项目落地开辟“绿色通道”，实现项目建设的重大突破。加强软环境建设。在梳理和集成现有政策手段基础上，制定新一轮鼓励产业转型发展的政策意见。加强知识产权保护，严厉打击非法侵权行为。优化政府行政服务，大力提高办事效率，降低商务成本，营造最具竞争力的发展环境，促进更多项目在无锡加快落地。

　　(四)深入推进改革开放，充分激发城市发展活力

　　加大改革攻坚力度，广泛利用全球资源，以改革开放为动力，突破发展难题，营造充满活力的城市发展环境。积极推进重点领域改革。大力推进投融资体制改革，鼓励社会资本参与政府建设项目投资，增加股权投资、上市融资等直接融资份额，大力支持民间资本办社会事业，形成投资主体多元化、融资渠道多样化的城市建设投融资新格局。推进文化演艺团体改革，鼓励面向市场，走向社会。深化管办分离改革，有效整合市属旅游、园林、文化、体育等领域经营性资源，按照企业化、市场化运行要求，探索建立新的法人治理结构。进一步落实促进民营经济健康发展的政策措施，拓展民间资本投资渠道，提升民营企业整体素质。加快利用外资步伐。围绕全市产业转型发展需求，努力招引一批重大外资项目，提升引进外资层级。健全激励制度，加强对利用外资的任务考核。进一步加强与国际大公司合作，大力提高新兴产业和现代服务业利用外资比例。鼓励外资以参股、并购等方式参与我市企业兼并重组。抢抓无锡-台湾直航效应，进一步拓展“台资高地”优势。大力开拓国际市场。努力稳定外贸出口，支持外贸外资企业抢抓订单，奋力开拓多元市场，千方百计稳定和扩大市场份额。不断提高一般贸易、服务贸易、“两自”产品出口比重。组织并推动企业积极参加展会或展销活动，引导有条件的企业构建全球市场营销体系。积极实施“走出去”战略，鼓励企业收购国外研发机构和高科技企业。加强口岸开放建设，开通无锡至香港、日本全货机，推动中国邮政速递长三角集散中心开工建设，启用海关国际快件监管中心。推动开发区转型升级。强化开发区功能提升，使之切实成为开放窗口、创新高地和项目集聚地。加强对各类开发区发展的统筹协调，放大江阴、锡山开发区升格为国家级开发区效应，加快功能载体建设，促进重大项目引进。积极推进宜兴国家环保科技工业园实行体制单列，无锡新区申报综合保税区，无锡经济开发区、宜兴开发区、江阴临港开发区申报国家级开发区及海关特殊监管区。

　　(五)加强城市建设管理，提升现代化城市功能

　　完善城市规划，突出重点，提高水平，加快推进现代化滨水花园城市建设。大力提升中心城市功能。以推进历史街区和重点片区建设为抓手，加快实施中心城区旧城改造，提升商贸休闲与综合服务功能，努力打造品质精致、功能完善的城市中心区。有序推进以太湖新城为重点的“五城”建设，增强集聚辐射能力。积极推进锡澄、锡宜之间在产业布局、基础设施、公共服务等方面的有机衔接，提升区域发展的整体合力。完善基础设施支撑体系。继续完善市内路网结构，打通关键节点，改善对城市交通有重大影响、对相关片区有重大作用的区域性主干道路的通行条件。加快地铁1、2号线建设，积极做好运营筹备工作。加快推进苏锡常南部高速公路、锡太高速公路等快速通道的前期工作，继续完善京沪高铁无锡东站综合功能，大力推进无锡(江阴)港、苏南(硕放)国际机场二期工程、宁杭铁路无锡段建设，完善城市对外综合交通体系，巩固和提升无锡作为区域性交通枢纽的城市地位。注重加强城市管理。完善城市管理目标体系，坚持工程科学概算，加大管理投入，提高重点基础设施的工程质量和使用效率。以市容秩序和环境卫生综合整治为抓手，着力解决市容环境难点和显见性问题。优化中心城区管理体制，注重发挥市(县)、区作用，加快建设网格化、精细化、长效化的城市管理体系，实现由执法管理为主向管理与服务并重转变。加快城乡发展一体化进程。以深化城乡“六个一体化”为主要抓手，以发展农村经济、增加农民收入为中心任务，以争创“幸福镇村”活动为工作重点，全面落实强农惠农富农各项政策措施。加快完善农村基础设施，大力推进村庄环境整治，着力改善农村生产生活条件，不断促进城乡之间要素自由流动、资源均衡共享、产业协调发展。全年建成通车农村公路150公里，改建农村危旧桥梁150座，实施一批重要农村水利工程，分两年完成7000个村庄整治任务，使城乡一体发展成为新时期无锡发展的特色和优势。

(六)着力提升环境品质，加快建设生态宜居城市

　　按照建设资源节约型、环境友好型社会的综合改革试点要求，深入推进生态文明先驱城市建设，加快改善生态环境整体质量，环境质量综合指数稳定达到90。扎实推进节能减排。落实化工、黑色金属冶炼及压延加工业、琉璃瓦和热电整合整治三年行动计划，加快淘汰落后产能。大力发展清洁能源，推进天然气储备基地建设，加快实施市区热电厂煤改气项目，力争建成区成为清洁能源区。推进电力、钢铁、水泥行业脱硫脱硝工程建设，确保完成年度减排任务。进一步强化太湖整治。加强控源截污，完成200公里污水主管网建设，全市城镇生活污水处理率达到92%，农村村庄生活污水治理覆盖率超过39%。城市污水处理厂在实现一级A排放基础上实施尾水生态净化和综合利用。完成走马塘拓浚延伸工程，启动建设新沟河工程，做好新孟河、望虞河西岸控制工程前期工作。完成太湖梅梁湖生态清淤200万立方米，加快贡湖湾湿地二期、长广溪湿地二期建设。全面改善环境质量。深入开展大气污染治理，着力控制建筑扬尘和汽车尾气排放。强化固体废弃物监管，推进污水处理厂污泥安全处置。整治交通和建筑噪声，不断改善城市声环境质量。巩固国家森林城市建设成果，完成植树造林3.5万亩，市区新增绿地500万平方米，全市林木覆盖率达到25.3%以上。狠抓资源集约利用。提高用地准入门槛，建立节约集约用地保证金制度，深入推进盘活存量土地专项行动。推进节水型社会建设，实行取水总量控制和用水定额管理，加快实施用水阶梯式计价制度和水资源有偿使用。进一步扩大节能和环境标志产品政府采购范围，鼓励节能、节水产品和再生利用产品生产。

　　(七)保障和改善民生，繁荣发展社会事业

　　以完善民生保障体系建设为重点，统筹推进各项社会事业协调发展。不断提高城乡居民收入。拓宽就业、创业、投资、社保及帮扶的增收渠道，大力开发就业岗位，加大创业扶持力度，促进农民经营性收入、财产性收入比重的不断提高。新增城镇就业10万人以上。稳步提高企业退休人员基本养老金和居民最低生活保障标准。重视关心中低收入者和困难群体，促进其生活的持续改善。抓好市场保供稳价。加快蔬菜基地建设，建立猪肉长效储备机制。加强农贸市场建设，开展市场环境专项整治。深化肉菜流通追溯体系建设，覆盖扩大到市区50%以上的农贸市场。加强价格监测预警和流通领域市场监管。进一步完善社会保障制度。重点推进农民工、非公经济组织从业人员、灵活就业人员和自由职业者参加社会保险，全市养老保险扩面10万人，医疗、失业保险扩面各4万人。提高城镇职工和居民基本医疗保险政策范围内住院费用基金支付比例，市区取消职工医疗保险最高支付限额，提高市、区两级财政对居民医保补助标准。落实低保增长机制，提高社会救助水平。市区居家养老服务机构全覆盖，全市新增机构养老床位3000张以上。改善居民居住条件。大力推进以公租房为主的住房保障体系建设，全面完成省下达的保障房建设任务。加快旧住宅区综合整治改造，年内完成150万平方米。完成中心城区棚户区危旧房改造40万平方米、城中村改造180万平方米，征地拆迁安置房竣工面积500万平方米。全面推进教育发展。提高学前教育发展水平，加快推进义务教育高位均衡发展示范区建设，实现全市义务教育现代化学校全覆盖，大力推进高中教育优质特色发展。深化职业教育综合改革，完善无锡职教园规划和管理体制。积极引进高等教育资源，推动中外合作办学。深入实施“校安工程”，切实加强学校安全管理。大力加强文化建设。繁荣发展公益文化事业，建立覆盖城乡的公共文化服务体系，健全公共图书馆、文化馆(站、室)免费开放制度，实现三级公共文化设施全年无休。推进地方历史文化的挖掘和宣传，加强鸿山国家考古遗址公园和阖闾城大遗址的保护与建设，深入推进历史文化街区和古村落保护利用，实施清名桥、惠山古镇中国历史文化名街及荡口、礼舍、长泾国家历史文化名镇村的品牌提升工程，积极推进中国大运河申报世界文化遗产示范工程，启动惠山祠堂申遗工程，支持江阴国家历史文化名城申报。积极创建国家公共文化服务体系示范区。争创国家全民健身示范城市，办好世界斯诺克无锡精英赛和环太湖国际公路自行车赛等品牌赛事，提升无锡城市影响力和知名度。加快医疗卫生事业发展。实施新一轮医药卫生体制改革。大力推进社区卫生服务站规范建设。推进卫生信息化进程，加大卫生人才培养和医学科技进步工作力度。加快市精神卫生中心住院病房大楼等重点医疗项目建设，市中医院易地建设项目、市三院门急诊楼年内投入使用。切实加强对食品、餐饮、药品、卫生等行业监管，强化社会监督，努力建设食品药品安全放心城市。

　　(八)加强和创新社会管理，促进社会和谐稳定

　　把社会管理作为新形势下政府服务群众的重要内容，通过管理与服务的改进完善，努力实现好、发展好广大人民的根本利益。凝聚社会发展合力。重视加强理想、道德、信念教育，强化正面宣传导向，弘扬“尚德务实、和谐奋进”的无锡城市精神，推进文明城市建设，提升城市形象，促进市民素质不断提高，增强无锡发展的向心力和凝聚力。优化人口管理服务。继续稳定低生育水平，合理调控人口规模。完善人口综合信息系统，积极稳妥推进户籍管理制度改革。重视加强老年工作。推进流动人口基本公共服务均等化，加大特殊人群动态管理服务力度，深入开展争创人口综合服务示范社区活动。深化基层社区管理。巩固城乡社区扁平化管理全覆盖成果，构建完善社区服务体系，全面完成社区事务工作站和农村综合服务中心建设，加强新建小区的社居委建设。积极推进社区、社会组织、社工互动。突出加强安全生产管理。强化队伍建设，推进企业安全生产标准化，全面落实安全生产责任制，着力夯实基层基础工作，突出抓好重点领域和薄弱环节监管，切实加强安全生产考核与责任追究，坚决遏制重特大事故发生，实现安全生产形势的根本好转。全力促进社会和谐稳定。建立维护社会稳定工作机制，实现稳评工作全覆盖，完善市、区、街道、社区四级调解组织，强化应急处置预案体系。高度重视信访工作，拓宽社情民意表达渠道。加强公安队伍建设，提高突发事件的预防预警和应急处置能力。加大社会治安综合治理力度，完善社会治安防控体系，严密防范、依法打击各类违法犯罪活动。做好民族、宗教工作。深入开展“双拥”活动，支持驻锡解放军和武警部队建设，努力营造社会和谐共进的良好发展环境。

　　切实加强政府自身建设

　　完成今年经济社会发展的繁重任务，政府工作责任重大。必须发扬开拓创新、求真务实的精神，切实履行职责，强化服务，努力建设人民满意的服务型政府。

　　全面提高依法行政水平。增强法制观念和民主意识，严格规范行政执法行为，大力推进政务公开，完善行政权力网上公开透明运行机制。充分尊重和发挥好人大代表和政协委员作用，认真办理人大代表和政协委员的议案、建议和提案，自觉接受人大法律监督和政协民主监督，主动接受群众监督和舆论监督。不断提升政府行政效能。机关工作人员尤其是领导干部要加强学习，注重研究新情况新问题，着力提高城市管理、社会管理和群众工作的本领，探索在市场紧缩条件下解决资金、资源及管理创新等方面的有效路径，以创造性工作破解发展中的难题。推进新一轮行政审批制度改革，优化管理流程，切实兑现向人民的承诺。创新服务手段，提升服务效能，向基层进一步下放管理权限，推行网上行政审批，进驻市行政服务中心审批事项中，列入网上审批的75%事项建立网上预审功能。大力弘扬求真务实作风。全面开展“作风建设年”活动，深入推进领导干部下基层，加强对企业、农村和社区的主动服务、超前服务，帮助克服困难。进一步改进工作作风，拿出更多时间深入基层、深入群众，多做打基础、利长远的事，多做解民忧、惠民生的事，坚决反对做表面文章、搞形式主义。加强机关绩效管理，不断提高机关工作效能。领导干部要带头脚踏实地、兢兢业业地做好各项工作，保持高尚健康的生活情趣和严谨正派的生活作风。着力加强政府廉政建设。要完善惩防体系建设，全面落实“一岗双责”，严格执行廉政建设各项规定，加强对权力运行的监督制约，切实解决损害群众利益的突出问题。加强审计和监察工作，强化重大工程项目的审计监督，打造政府项目的“阳光工程”。各级政府带头厉行节约，加强对部门预算与支出的规范管理，严格控制行政费用支出，把更多的物力、财力用在率先基本实现现代化的事业上，用在人民群众最迫切需要解决的问题上，努力建设节约型政府和廉洁政府，树立起全市人民所信赖的良好形象。各位代表，率先基本实现现代化，是全体无锡人民的共同期盼；扎实做好今年工作，是顺利实现这一目标的坚实基础。让我们紧密团结在以胡锦涛同志为总书记的党中央周围，按照省委省政府的决策部署，在中共无锡市委的正确领导下，紧紧围绕市第十二次党代会和市委十二届二次全会的部署要求，凝心聚力，扎实工作，全面完成今年各项任务，为把无锡的明天建设得更加美好而努力奋斗！
